# CD11b deficiency suppresses intestinal tumor growth by reducing myeloid cell recruitment

**DOI:** 10.1038/srep15948

**Published:** 2015-11-03

**Authors:** Qian-Qian Zhang, Xi-Wen Hu, Yi-Long Liu, Zhi-Jin Ye, Yi-He Gui, Da-Lei Zhou, Cui-Ling Qi, Xiao-Dong He, Honglin Wang, Li-Jing Wang

**Affiliations:** 1 Vascular Biology Research Institute, School of Basic Course, Guangdong Pharmaceutical University, Guangzhou 510006, China; 2 Institute of Immunology, Institute of Medical Sciences, Shanghai Jiao Tong University School of Medicine, Shanghai 200025, China

## Abstract

Mac-1 (CD11b) is expressed on bone marrow-derived immune cells. CD11b binds to ligands to regulate leukocyte adhesion and migration across the endothelium or epithelium. Here, we employed *CD11b* knockout mice and an *Apc*^*Min/*+^ spontaneous intestinal adenoma mouse model to clarify the function of CD11b in intestinal tumorigenesis. We showed that CD11b deficiency may contribute to the inhibition of myeloid cell trafficking to the tumor microenvironment and inactivated Wnt/β-catenin pathway to suppress tumor growth. This effect was partly mediated by inhibiting the myeloid cell-mediated decrease in TNF-α secretion, which inhibits the recruitment of myeloid-derived suppressor cells to the tumor microenvironment and subsequently induces IFN-γ and CXCL9 production. This work provides evidence for the mechanism by which CD11b may function as an important oncogene and highlights the potential of CD11b as a therapeutic target in CRC.

Colorectal cancer (CRC) is the most common cause of cancer death world-wide. The accumulated molecular genetic alterations that underlie the development of CRC are well characterized^1^,^2^. However, the role of the tumor microenvironment during CRC tumorigenesis is not completely understood^2^,^3^. The development of CRC is a multistep process that involves interactions between the tumor cells and the host microenvironment. The tumor microenvironment consists of tumor cells, carcinoma-associated fibroblasts, leukocytes, blood, lymphatic vascular endothelial cells and the extracellular matrix, which contribute to tumor progression^4^,^5^. Moreover, leukocytes progressively accumulate in the tumor microenvironment, and immune reactions accompany all stages of CRC^6^,^7^,^8^. Among the leukocytes that infiltrate the tumor microenvironment, bone marrow-derived myeloid cells predominantly accumulate in tumors and stimulate cancer initiation, progression and resistance to anti-cancer therapy^4^,^7^,^9^. However, the regulatory mechanism by which myeloid cell infiltration into the tumor microenvironment regulates tumor growth to be further clarified.

CD11b (Mac-1, αMβ2) is the α-subunit of the predominant β2 (CD18) integrin adhesion molecule, which is expressed on the surface of the myeloid cells^10^,^11^. On bone marrow-derived immune cells, CD11b is responsible for facilitating cell adhesion to and transmigration across the endothelium or epithelium and traffic to the inflammation sites to mediate the inflammatory response^7^,^10^,^12^,^13^,^14^,^15^. A recent report indicated that Mac-1 inhibition can reduce myeloid cell recruitment to the tumor environment and thereby enhance the tumor’s response to radiation^11^. Although CD11b has been widely implicated in myeloid cell adhesion and migration, the biological significance and molecular mechanisms of the role of CD11b in myeloid cell infiltration into the tumor microenvironment are largely undetermined.

Myeloid derived suppressor cells (MDSCs) are a population of immature myeloid cells that originate from the bone marrow. Several recent publications demonstrated that MDSCs are CD11b^+^Gr-1^+^ immunosuppressive cells that suppress T-cell activation and accumulate in the bone marrow, spleen and tumor sites in most patients and in tumor-bearing mice^16^,^17^. MDSCs consist of two major subsets of cells: cells with a granulocytic phenotype (Ly6G^+^Ly6C^low^) and cells with a monocytic phenotype (Ly6G^-^Ly6C^high^)^18^,^19^. Only the Ly6G^+^Ly6C^low^ granulocytic MDSCs have been shown to expand in most tumor models, and these cells may play an important role in the general process of angiogenesis and tumor progression^19^,^20^,^21^. Moreover, the number of circulating MDSCs was also significantly increased in cancer patients and correlated with the clinical cancer stage^19^. Recent studies demonstrated that myeloid cells in tumor hosts can be transported to the spleen, peripheral blood and tumor sites and produce cytokines and chemokines that activate MDSCs and allow them to infiltrate the tumor sites^17^. Therefore, we hypothesized that CD11b-deficient myeloid cells may likely regulate MDSCs infiltration into the tumor environment and thereby inhibit the angiogenesis and tumor growth of colorectal carcinoma.

Wnt/β-catenin signaling is constitutively activated in nearly all colorectal tumors and leads to a loss of E-cadherin and the nuclear translocation of β-catenin. This signaling pathway then modulates the expression of a broad spectrum of target genes to promote tumor cell proliferation. Inflammatory cells in CRC tumors have been shown to be associated with tumor progression. Moreover, cytokines that are secreted by the inflammatory cells may interact with the Wnt signaling pathway in the tumor microenvironment and lead to an accumulation of β-catenin in the nucleus^22^. However, the ability of MDSCs that infiltrate the tumor environment to activate Wnt/β-catenin signaling in CRC is unknown.

In this study, we used the *Apc*^*Min/*+^ CRC mouse model and Mac-1-deficient (*CD11b*^−/−^) mice to define the possible role and the underlying regulatory mechanisms of Mac-1 in intestinal tumorigenesis.

## Materials and Methods

### Reagents and antibodies

TNF-α was obtained from Peprotech (La Jolla, CA). Bromodeoxyuridine (BrdU) cell proliferation labeling reagent (RPN201, intraperitoneally injected with 0.1 mg/g mouse weight, 2 hours before killed) was obtained from GE Healthcare (Little Chalfond, UK). The following antibodies were used for Flow cytometry: anti-CD45-FITC (eBioscience, San Diego, CA), anti-CD11b-PC7 (BD Pharmingen, San Diego, CA), anti-Ly6C-BV421 (BioLegend, San Diego, CA), anti-Ly6G-PE-CF594 (BD Horizon, San Jose, CA). The primary antibodies were used for immunohistochemical (IHC), immunofluorescence (IF) analysis and Western blotting (WB) assays: and mouse anti-BrdU antibody (RPN202, diluted at 1:150 for IHC) was obtained from GE Healthcare (Little Chalfond, UK); rabbit anti-CyclinD1 (BM0771, diluted at 1:100 for IHC and 1:400 for WB), rabbit anti-CD34 (BA0532, diluted at 1:100 for IHC), rabbit anti-CD45 (BA3371, diluted at 1:100 for IHC) and rabbit anti-p65 (BA0610, diluted at 1:100 for IHC) were obtained from Boster (Wuhan, China); mouse anti-TNF-α (ab10863, diluted at 1:5000 for WB) was obtained from Abcam (Cambridge, UK); rabbit anti-IFN-γ (bs-0480R), rabbit anti-CXCL-9 (bs-2551R) (both diluted at 1:500 for WB), rabbit anti-CD11b (bs-1014R) and rabbit anti-Gr-1 (bs-2576R, diluted at 1:100 for IF) were obtained from Bioss (Beijing, China); mouse anti-Cytokeratin 8 (CK8, ZM-0310, diluted at 1:100 for IF) was purchased from ZSGB-BIO (Beijing, China); mouse anti-CD11b (Santa Cruz, USA, diluted at 1:100 for IF); mouse anti-E-cadherin (#610181, diluted at 1:100 for IHC and 1:5000 for WB) and mouse anti-β-catenin (#610154, diluted at 1:100 for IHC, and 1:2000 for WB) were purchased from BD Transduction Laboratories (Franklin Lakes, NJ); rabbit anti-GAPDH (#2118s, diluted at 1:2000 for WB) was purchased from CST (USA); rabbit anti-pp65 (Ser276, sc-101749, diluted at 1:500 for IHC) was obtained from Santa Cruz Biotechnology Inc. (USA).

### Patients and tissue samples

A total of 10 cases of colonic tumor tissues and their matched non-tumorous colonic tissues were collected from the Department of Pathology, The Third Affiliated Hospital of Sun Yat-Sen University. Written informed consent from each patient was obtained prior to the initiation of this study. Pathologic diagnosis was performed by two independently pathologists. All methods were performed in accordance with the guidelines approved by the Ethics Committee of Medicine, Guangdong Pharmaceutical University.

### Animals and treatment

C57BL/6 (C57) mice were purchased from the Guangdong Medical Laboratory Animal Center. *APC*^*Min/*+^ mice (strain: C57BL/6J-*Apc*^*Min*^/J, stock number: 002020) and *CD11b*^*−/−*^ mice (strain: B6.129S4-*Itgam*^*tm1Myd*^/J, stock number: 003991) on the C57BL/6J background were purchased from the Jackson Laboratory (Bar Harbor, ME). *APC*^*Min/*+^;*CD11b*^*−/−*^ mice were obtained by crossbreeding the *APC*^*Min/*+^ mice and *CD11b*^*−/−*^ mice to the F2 generation. The founder mice were viable and exhibited normal growth. Genotyping was performed by PCR using genomic DNA prepared from mouse tail according to the genotyping protocol. The primers for *CD11b* genotyping were 5′- TAG GCT ATC CAG AGG TAG AC-3′ (for the wild-type and targeted alleles); 5′- CAT ACC TGT GAC CAG AAG AGC-3′ (for the wild-type allele); and 5′- ATC GCC TTC TTG ACG AGT TC-3′ (for the targeted allele). The primers for *APC*^*Min/*+^ genotyping were 5′- GCC ATC CCT TCA CGT TAG-3′, 5′- TTC CAC TTT GGC ATA AGG C-3′ and 5′- TTC TGA GAA AGA CAG AAG TTA-3′ (for both the wild-type and targeted alleles). Eleven mice from each group were used for the studies, unless otherwise indicated. All mice (15-week old) that were used for analyses were maintained in a climate-controlled room at a temperature of 24 ± 2°C and a relative humidity of 60 ± 5% under a 12-h light/dark cycle. All surgical procedures were performed under diethylether anesthesia. BrdU (0.1 mg/g body weight) was intraperitoneally injected into the mice, which were sacrificed 2 hours later. All experiments were performed in accordance with the protocols approved by the Ethics Committee of the Center of Laboratory Animals at Guangdong Pharmaceutical University.

### Cell Culture

The human HCT-116 and mouse CT-26 colorectal carcinoma cell lines were obtained from the cell bank of the Chinese Academy of Sciences (Shanghai, China). The cells were maintained in Dulbecco’s Modified Eagle’s Medium (DMEM, GIBCO) supplemented with 10% fetal bovine serum (FBS), 100 U/mL penicillin and 100 μg/mL streptomycin and incubated in a humidified chamber containing 5% CO_2_ at 37 °C. TNF-α was added to the cells at a dose of 50 ng/mL for 48 hours, and the total proteins were prepared for an immunoblotting analysis.

### Isolation of myeloid cell and co-culture

The single suspension of bone marrow derived all nucleated cells from femurs and tibias was collected and maintained in DMEM supplemented with 100 μg/mL PHA, 10% FBS, 100 U/mL penicillin and 200 μg/mL streptomycin and incubated in a humidified chamber containing 5% CO_2_ at 37 °C for 2 days.

CT-26 cells and HCT-116 cells were seeded into 24-well plates at a density of 8 × 10^4^ per well and 2 × 10^5^ per well respectively without FBS for 24 hours, and then re-added FBS and co-cultured with myeloid cells for 24 hours. Myeloid cells were loaded into the upper compartment of the transwell chambers at a density of 1:10 (myeloid cell: tumor cell) with or without TNF-α antibody (mouse anti-TNF-α, 1:500, BA0131, Boster, China). Then the tumor cells were collected for further detection.

### Analysis of intestinal tumors

After the *APC*^*Min/*+^ mice and *APC*^*Min/*+^*;CD11b*^*−/−*^ mice were sacrificed, the entire gastrointestinal tract was excised and separated into the colon and 3 segments of the small intestine: proximal, medial, and distal. All regions were opened longitudinally, flattened between sheets of filter paper, immersed in 10% phosphate-buffered formalin, and then stained with 10% methylene blue. The tumor numbers and sizes were determined using dissecting microscope (OLYMPUS, Japan), and the tumor volume (V) was calculated according to the following equation: V = (L × W^2^) × 0.5236 (L: length; W: width). The intestinal neoplasias were classified using microscope as described previously^23^ (Supplementary Figure 1).

### Histological and immunohistological staining

The intestinal tissues were fixed in neutral-buffered 10% formalin solution, embedded in paraffin, and sectioned to a thickness of 3 μm. Hematoxylin & eosin (H&E), immunohistochemical (IHC) and immunofluorescent (IF) staining for BrdU, CD34, β-catenin, E-cadherin, Cyclin D1, CD45, CD11b, CK8 and Gr-1 were performed as previously described^24^,^25^. The sections were then observed under a scanning confocal microscope (Leica, Germany).

### Microvessel Density

Microvessel density (MVD) was recorded as the number of point counts of endothelial cells with the specific antibody to CD34 per field at × 200 magnification. Ten fields were randomly selected in a section of tumors were examined. MVD counts were recorded independently by two observers in sections from three mice of each group.

### Immunoblotting

The intestines were sliced longitudinally, and the macroscopic tumors were cut off from the intestines. The total proteins from the tumors and cells were prepared using RIPA buffer, and immunoblotting assays were performed as previously described^26^.

### Flow cytometry (FACS)

A single cell suspension of blood cells, bone marrow cell, splenocytes or tumor digests that had been treated as described above was subjected to flow cytometry using the following MDSC surface markers: CD45, CD11b, Ly6C, and Ly6G. To analyze the inflammatory cell infiltrates in the tumor tissue, the tumors were mechanically dissociated on a wire mesh by crushing with the plunger of a 10-mL syringe and then incubated in tissue-digestion buffer at 37 °C for 25 min. The cells were filtered through 70-μm nylon strainers (BD Biosciences, Bedford, MA), stained with specific antibodies and analyzed by flow cytometry. The FACS data were acquired using a Beckman Coulter Gallios flow cytometer and were analyzed using the FlowJo software package (Tree Star, Ashland, OR, USA).

To detect the cell cycle progression, the tumor cells in co-culture system were collected and fixed the cells with 75% ethanol for 40 min at 4 °C, centrifuged, washed twice in phosphate buffered saline, and stained with PI solution (#550825, BD Biosciences, USA) at 37 °C for 15 min. The analysis was performed using a FACS Calibur flow cytometer (Becton Dickinson) and analyzed using the Modfit software, version 3.0 (Verity Software House).

### Real-time quantitative PCR arrays

The total RNA was extracted from the blood or spleen of mice using TRIzol reagent (Invitrogen) according to the manufacturer’s instructions. The total RNA (500 ng) was reverse transcribed using an PrimeScript^TM^ RT Reagent Kit (TaKaRa, Japan), and the real-time quantified PCR was performed on a LightCycler480 PCR machine (Roche) using the SYBR® Premix Ex Taq™ II (Tli RNaseH Plus) PCR Kit (TaKaRa, Japan), to the manufacturer’s instructions. The data were analyzed using the 2^−∆∆CT^ methodology as described^27^.

### Enzyme-linked immunosorbent assay (ELISA)

Serum was collected from the *APC*^*Min/*+^ mice and *APC*^*Min/*+^*;CD11b*^*−/−*^ mice, and the levels of TNF-α were analyzed using a Mouse/Rat TNF-α Valukine ELISA Kit (1 KT) (VAL609, R&D Systems, USA), according to the manufacturer’s instructions. Each sample was measured in triplicate.

### Statistical analysis

The data are presented as the mean ± standard deviation (SD) and the differences between groups were analyzed using a Student’s *t* test. Differences were considered statistically significant at *P* < 0.05. The protein expression levels in the IHC slices were determined by measuring the cumulated integrated optical density (IOD) using IPP software (Media Cybernetics, Inc., USA). The densitometric analysis of the immunoblotting bands was performed using Quantity One software (Bio-Rad, USA), and the protein band intensities were quantitated and normalized to those of GAPDH.

## Results

### Intestinal tumor development is accompanied by infiltrating myeloid cells

**Figure 1:**
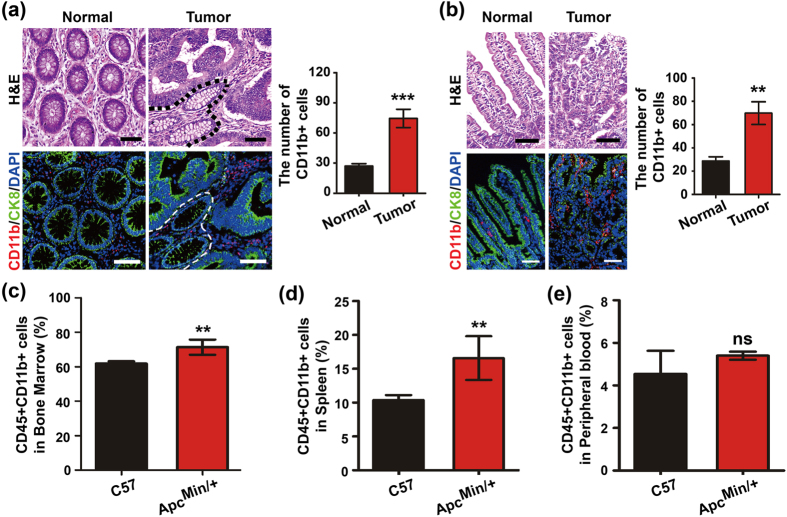
Intestinal tumor development is accompanied by infiltrating CD11b^+^ myeloid cells. The number of CD11b^+^ myeloid cells increased in tumor tissues compared with the non-cancerous colonic tissues in CRC patients (**a**). The morphology of the colonic tissues was examined by H&E staining. The myeloid cell infiltration was detected by IF in the tumor tissues of CRC patients. CD11b^+^ myeloid cells were increased in tumor tissues compared with the normal small intestine tissues of *Apc*^*Min/*+^ mice (**b**). The morphology of the colonic tissues was examined by H&E staining. The myeloid cell infiltration was detected by IF in the tumor tissues of *Apc*^*Min/*+^ mice. The numbers of CD11b^+^ myeloid cells in the bone marrow (**c**), spleen (**d**) and peripheral blood (**e**) were analyzed by FACS gated on CD45^+^ leukocytes. The results of H&E and IF staining are representative of 11 independent CRC patients or *Apc*^*Min/*+^ mice (all mice were 16-weeks-old). The statistical data are expressed as the means ± S.D. **P <* *0.01, and ns: *P *> 0.05. Scale bars: 50 μm.

Infiltrating inflammatory myeloid cells often reportedly heavily infiltrate solid tumors^7^,^28^. To determine the level of myeloid cell infiltration, we stained the tumors with a CD11b antibody to detect myeloid cells using immunofluorescence analysis (IF) and further stained a single cell suspension of blood cells, bone marrow cell and splenocytes with CD11b antibody to detect myeloid cells (gated on CD45^+^ cells (all leukocytes)) using FACS assay^11^. To study the tumor microenvironment, we immunofluorenscently stained the human tumor tissues (Tumor) and their matched surrounding non-cancerous colonic tissues (Normal) with CD11b antibody. We observed that the levels of the CD11b^+^ myeloid cells were increased in the tumor tissues compared with the non-cancerous colonic tissues (Fig. 1a). The *Apc*^*Min/*+^ mouse is a model for the development of CRC. This transgenic mouse model has been widely used to study the development of intestinal tumorigenesis^29^. We examined the infiltration of myeloid cells in the spontaneous adenomatous tissues of *Apc*^*Min/*+^ mice by IHC staining. Many CD11b^+^ myeloid cells had infiltrated the mesenchyme of the tumor region (Fig. 1b). In addition, we detected the number of CD11b^+^ myeloid cells (gated on CD45^+^ cells) in the bone marrow, spleen and peripheral blood by FACS to determine the myeloid cell contribution to intestinal tumorigenesis. With the exception of the peripheral blood, the CD11b^+^ myeloid cell populations were substantially enriched in the bone marrow and spleen of the *Apc*^*Min/*+^ mice compared with the C57 mice (Fig. 1c–e). These results suggest that CD11b^+^ myeloid cells are activated during CRC development.

### CD11b deficiency inhibits intestinal tumor growth *in vivo*

**Figure 2:**
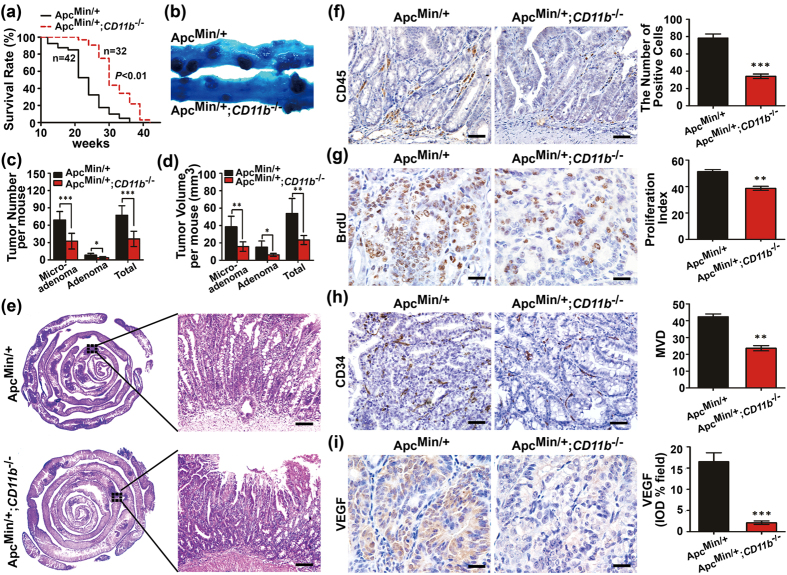
CD11b deficiency inhibits intestinal tumor growth. The small intestines from the *Apc*^*Min/*+^ and *Apc*^*Min/*+^*; CD11b*^*−/−*^ mice were examined macroscopically after methylene blue staining (**a**). CD11b deficiency significantly inhibited the number (**b**) and size (**c**) of the tumors, and prolonged survival (**d**). *Apc*^*Min/*+^: n = 11, *Apc*^*Min/*+^*;CD11b*^*−/−*^: n = 16 (**b–d**). A holistic view of the histological lesions in the intestines of the *Apc*^*Min/*+^ mice and *Apc*^*Min/*+^*;CD11b*^*−/−*^ mice as observed on the “whole intestines” under a microscope (**e**). CD11b deficiency resulted in a marked decrease in the infiltration of CD45^+^ leukocytes (**f**); in cell proliferation, as indicated by attenuated BrdU uptake in the tumor tissues of *Apc*^*Min/*+^*;CD11b*^*−/−*^ mice (**g**); and in angiogenesis, as indicated by the decreased expression of CD34 (**h**) and VEGF (**i**). The IHC results were quantitated using the IPP software (**f–i**, right panel). The results of the methylene blue staining and IHC staining are representative of 11 independent mice (all mice were 16–weeks-old). The statistical data are expressed as the mean ± S.D. **P < 0.01, and ***P < 0.001. Scale bars: 100 μm (**e**), 50 μm (**f**,**h**) and 20 μm (**g**,**i**).

β2-integrin and CD11b expression on bone marrow-derived immune cells regulates leukocyte adhesion and migration to the tumor microenvironment to mediate the inflammatory response^7^,^15^,^30^. The *Apc*^*Min/*+^ mice were crossed with *CD11b*^*−/−*^ mice to generate *Apc*^*Min/*+^;*CD11b*^*−/−*^ mice (Supplementary Figure 2a–d), which were used to further confirm the effect of CD11b ^+^ myeloid cells infiltration into the tumor microenvironment on the intestinal tumorigenesis. As shown in Fig. 2a, the *Apc*^*Min/*+^;*CD11b*^*−/−*^ mice were viable and survived significantly longer than the *Apc*^*Min/*+^ mice. A macroscopic examination showed that the *Apc*^*Min/*+^; *CD11b*^*−/−*^mice exhibited a significantly decreased the number of tumors and average tumor volume in the small intestine but not the colon compared with the *Apc*^*Min/*+^ mice (Fig. 2b–d and Supplementary Figure 2e). A holistic view of the intestines indicated fewer highly condensed nests of neoplastic cells in the *Apc*^*Min/*+^;*CD11b*^*−/−*^ mice compared with the *Apc*^*Min/*+^ mice (Fig. 2e, left panel). Moreover, we also observed the development of invasive carcinoma in the intestines of 15-week-old *Apc*^*Min/*+^ mice, albeit less frequently. However, invasive carcinoma was not observed in the *Apc*^*Min/*+^;*CD11b*^*−/−*^ mice at the same age (Fig. 2e, right panel).

Reports indicated that myeloid cells infiltrate the tumor microenvironment can support tumor growth and promote tumor angiogenesis^4^,^17^. The results showed that depletion of CD11b^+^ cells in the *Apc*^*Min/*+^ mice abrogated leukocyte cell infiltration in the tumor microenvironment but not in the adjacent normal villus tissue (Fig. 2f and Supplementary Figure 2g). We further detected tumor cell proliferation and angiogenesis by IHC staining. Most tumors in the experimental CD11b-deficient mice displayed significantly fewer BrdU-positive cells (Fig. 2g) and CD34 point counts as well as significantly reduced VEGF expression (Fig. 2h,i). These data suggest that the CD11b-deficient myeloid cells significantly inhibited the growth of intestinal tumors.

### CD11b deficiency inactivates the Wnt/β-catenin pathway during intestinal tumorigenesis

**Figure 3:**
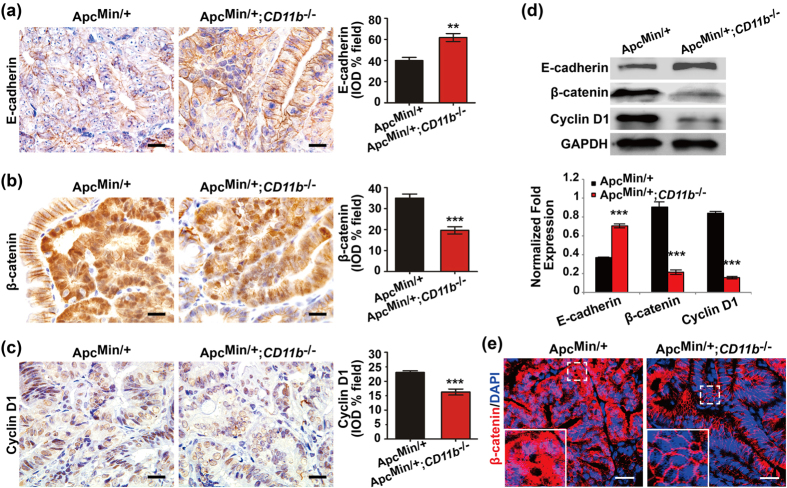
CD11b deficiency inactivated the Wnt/β-catenin pathway. The expression of E-cadherin, β-catenin and cyclin D1 was examined in the tumor tissues of the *Apc*^*Min/*+^ and *Apc*^*Min/*+^*;CD11b*^*−/−*^ mice by IHC (**a**) and immunoblotting (**b**), and the IHC results were quantitatively determined using IPP the software and expressed as the means ± S.D. The subcellular location of β-catenin was examined in the tumor tissues of the *Apc*^*Min/*+^ and *Apc*^*Min/*+^*;CD11b*^*−/−*^ mice by IF (**c**). All IHC and IF staining in the mouse tissues represent 11 independent mice (all mice were 16-week-old). The IHC results were quantified using the IPP software and are expressed as the means ± S.D. **P < 0.01, ***P < 0.001. Scale bars, 20 μm (**a**–**c**) and 25 μm (**e**).

The canonical Wnt/β-catenin signaling pathway is critical for the homeostasis and neoplastic transformation of the intestinal tract^31^,^32^. *APC* mutation results in the activation of the Wnt pathway. Constitutively active Wnt/β-catenin signaling is associated with CRC initiation, and leads to an accumulation of β-catenin in the nucleus and a loss of E-cadherin. Whether CD11b-deficient myeloid cells that infiltrate the tumor microenvironment inhibit intestinal tumorigenesis by inactivating the Wnt/β-catenin signaling has not yet been determined. Compared with the *Apc*^*Min/*+^ mice, IHC staining (Fig. 3a–c and supplementary Figure 3) and immunoblotting assay (Fig. 3d) indicated a significant increase in the expression of E-cadherin and decreased expression levels of β-catenin and cyclin D1, the downstream target of the Wnt/β-catenin pathway, in the tumor tissues but not in the adjacent normal tissue of the *Apc*^*Min/*+^;*CD11b*^*−/−*^ mice. Decreased nuclear translocation of β-catenin was observed in the tumor cells of the *Apc*^*Min/*+^;*CD11b*^*−/−*^ mice by IF staining (Fig. 3e). These results indicate that activated Wnt/β-catenin signaling was partially inhibited by deficient CD11b^+^ myeloid cells infiltration into the tumor microenvironment.

### CD11b deficiency inhibits intestinal tumor growth by reducing TNF-α release

**Figure 4:**
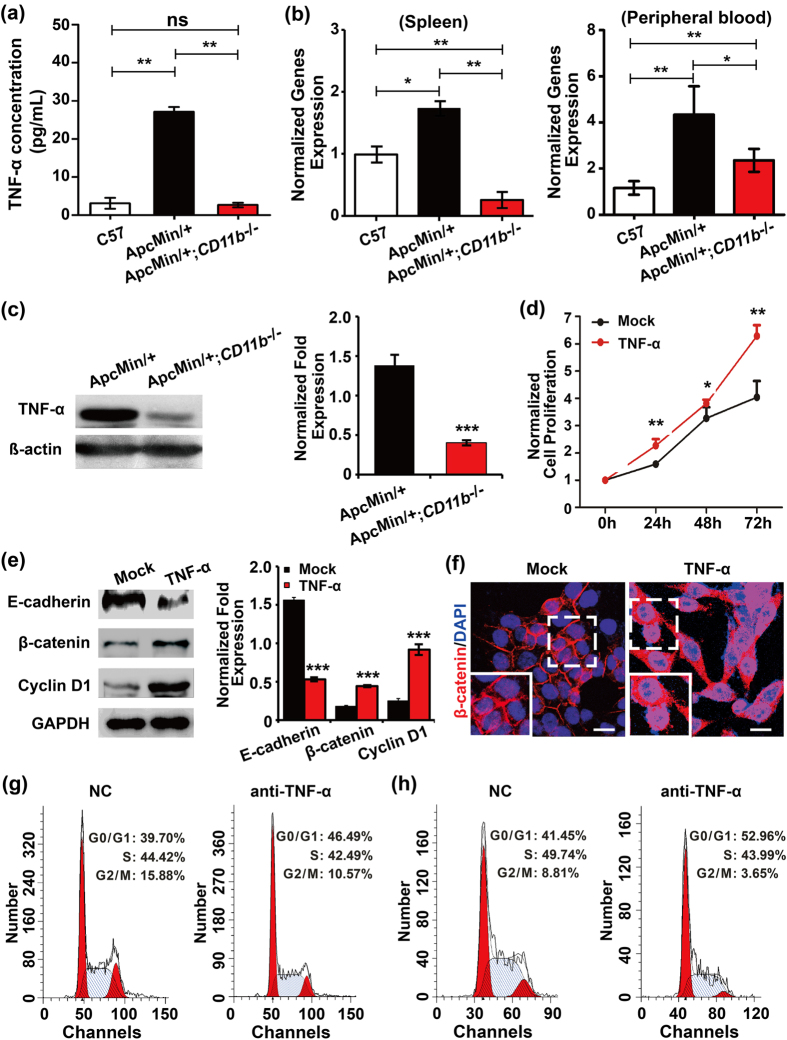
CD11b deficiency inhibits tumor growth by suppressing TNF-α release. The concentration of TNF-α in the peripheral blood of the C57, *Apc*^*Min/*+^ and *Apc*^*Min/*+^*;CD11b*^*−/−*^ mice was examined by ELISA (**a**). The mRNA level of TNF-α in the peripheral blood and spleen of the C57, *Apc*^*Min/*+^ and *Apc*^*Min/*+^*;CD11b*^*−/−*^ mice was examined by qRT-PCR (**b**). The proteins expression of TNF-α in the tumor tissues from the *Apc*^*Min/*+^ and *Apc*^*Min/*+^*; CD11b*^*−/−*^ mice was examined by immunoblotting (**c**). The protein band intensities were quantitated and normalized to those of GAPDH. TNF-α can significantly promoted cell proliferation (**d**) and Wnt/β-catenin activation (**e**) in HCT-116 cells (this cell line shows low Wnt/β-catenin signaling activity). TNF-α can also induce the nuclear translocation of β-catenin in HCT-116 cells (**f**), Scale bar: 25 μm. All results represent at least three separate experiments (**d–f**). Inhibit TNF-α arrested CT-26 cells (**g**) and HCT-116 cells (**h**) at the G1/S transition in myeloid cell and tumor cell co-culture system. The results are expressed as the means ± S.D. ns: *P *> 0.05, *P < 0.05, **P < 0.01 and ***P < 0.001.

Myeloid cells that infiltrate the tumor microenvironment stimulate cancer initiation, malignant progression and angiogenesis by releasing a number of potent pro-tumorigenic cytokines. Therefore, the Inflammatory Cytokines & Receptors PCR Array (#APM-011, SUPERARRAY, USA) was used to identify the cytokines that might affected by CD11b^+^ myeloid cells in CRC using a quantitative RT-PCR assay (supplementary Figure 4a). The results indicated that the CD11b-deficent tumor tissue exhibited a robust 1.36-fold decrease in TNF-α expression. We detected the concentration of TNF-α in the peripheral blood with an ELISA assay, and found that the TNF-α level in peripheral blood was up-regulated in the *Apc*^*Min/*+^ mice compared with the C57 mice. However, the increase in the TNF-α level was reversed in CD11b-deficient *Apc*^*Min/*+^ mice (Fig. 4a). The total RNAs of the peripheral blood and spleen were extracted, and the quantitative RT-PCR assay showed the same effect as the ELISA (Fig. 4b). TNF-α expression was also decreased in the tumor tissues of the *Apc*^*Min/*+^;*CD11b*^*−/−*^ mice compared with *Apc*^*Min/*+^ mice, as analyzed by immunoblotting (Fig. 4c). We further detected the effect of TNF-α on tumor growth and Wnt/β-catenin signaling activity in HCT-116 cells (this cell line shows low Wnt/β-catenin signaling activity) and found that TNF-α significantly induced cell proliferation (Fig. 4d) and promoted the activation of Wnt/β-catenin signaling by inhibiting the expression of E-cadherin and up-regulating the expression of β-catenin and cyclin D1 (Fig. 4e). Further confocal microscopy studies also confirmed that TNF-α significantly induced the nuclear translocation of β-catenin in HCT-116 cells (Fig. 4f). Meanwhile, we observed the expression of NF-κB, TNF-α downstream targets, and found that the expression of p65 showed no difference in the tumor tissues between the *Apc*^*Min/*+^*;CD11b*^*−/−*^mice and *Apc*^*Min/*+^ mice. However, the expression of pp65 (Ser276) was significantly inhibited in the tumor tissues of the *Apc*^*Min/*+^*;CD11b*^*−/−*^ mice compared with *Apc*^*Min/*+^ mice (Supplementary Figure 4b). Moreover, the myeloid cells were isolated from the bone marrow of C57 mice and co-culture with CT-26 and HCT-116 cells. Compared with the negative control (NC) group, CT-26 (Fig. 4g) and HCT-116 (Fig. 4h) cells significantly accumulated in the G0/G1 peak and arrested the cell cycle at the G1/S transition in TNF-α antibody treated co-culture system. These results demonstrated that CD11b deficiency suppressed tumor growth by reducing the levels of TNF-α secreted by myeloid cells.

### CD11b deficiency reduces MDSC recruitment in the tumor environment

**Figure 5:**
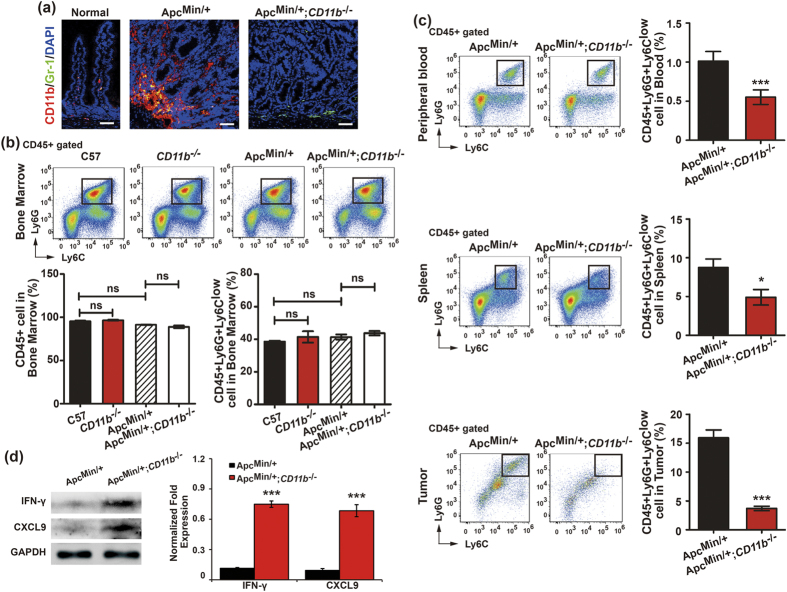
CD11b deficiency reduced myeloid-derived suppressor cells (MDSC) recruitment in the tumor environment. The accumulation of Gr-1^+^CD11b^+^ MDSC cells in the tumor microenvironment of the *Apc*^*Min/*+^ mice compared with the normal intestine tissues from the C57 mice; the Gr-1^+^ cells were decreased in the tumor microenvironment of the *Apc*^*Min/*+^*;CD11b*^*−/−*^ mice (**a**). The number of the Ly6G^+^Ly6C^low^ subset of MDSCs (gated on CD45^+^ cells) in the bone marrow of C57, *CD11b*^*−/−*^, *Apc*^*Min/*+^ and *Apc*^*Min/*+^*;CD11b*^*−/−*^ mice (**b**) and in the peripheral blood, spleen and tumor tissues of the *Apc*^*Min/*+^ and *Apc*^*Min/*+^*;CD11b*^*−/−*^ mice (**c**) was measured by FACS. The results of the IF staining and FACS represent of 11 independent mice (all mice were 16–weeks-old). The proteins expression of IFN-γ and CXCL9 in the tumor tissues from the *Apc*^*Min/*+^ and *Apc*^*Min/*+^*;CD11b*^*−/−*^ mice was examined by immunoblotting (**d**). The protein bands intensities were quantitated and normalized to those of GAPDH. The statistical data are expressed as the means ± S.D. *P<0.05, **P < 0.01 and ***P < 0.001. Scale bars: 50 μm (**a**).

The Ly6G^+^Ly6C^low^ subset of MDSCs exhibits pro-inflammatory activity and often enriched in tumor models^16^,^19^. Previous reports indicated that TNF-α is secreted by myeloid cells and enriched in the tumor microenvironment, which can drive MDSC accumulation^33^,^34^,^35^. Moreover, MDSCs can contribute to angiogenesis and facilitate tumor growth^17^. The Ly6G^+^Ly6C^low^ subset of MDSCs was significantly larger during CD11b+ myeloid cell-mediated tumor growth. We demonstrated that Gr-1^+^CD11b^+^ MDSCs accumulated in the tumor cells in the tumor microenvironment of the *Apc*^*Min/*+^ mice, but the number of Gr-1^+^ cells decreased in the tumor microenvironment of CD11b-deficient tumor-bearing mice (Fig. 5a). We further detected the difference in production of the Ly6G^+^Ly6C^low^ subset of MDSCs (gated on CD45^+^ cells) in the bone marrow of *CD11b*^*−/−*^ mice and *CD11b*^*−/−*^ tumor-bearing mice (wild type mice were used as controls) and found that the CD45^+^ leukocyte and granulocytic Ly6G^+^Ly6C^low^ MDSC populations did not differ in the bone marrow of the C57, *CD11b*^*−/−*^, *Apc*^*Min/*+^ and *Apc*^*Min/*+^;*CD11b*^*−/−*^ mice (Fig. 5b). However, CD11b deficiency significantly inhibited the number of granulocytic Ly6G^+^Ly6C^low^ MDSCs that were recruited to peripheral blood, spleen and tumor microenvironment in the *Apc*^*Min/*+^;*CD11b*^*−/−*^ mice compared with the *Apc*^*Min/*+^ mice (Fig. 5c).

MDSCs inhibit IFN-γ production by specifically inhibiting the CD8-mediated Ag-specific T cell response^36^,^37^. Moreover, IFN-γ can induce CXCL9 production^38^,^39^. Reports indicated that IFN-γ and CXCL9 can attenuate angiogenesis and tumor growth^40^,^41^,^42^. We further detected the expression of IFN-γ and CXCL9 in tumor site, and found that IFN-γ and CXCL9 were significantly up-regulated in the tumor tissues of the *Apc*^*Min/*+^;*CD11b*^*−/−*^ mice compared with the *Apc*^*Min/*+^ mice (Fig. 5d). These results demonstrate that CD11b deficiency suppressed tumor growth by reducing the amount of TNF-α secreted by myeloid cells and inhibiting MDSCs recruitment to the tumor microenvironment, which further prevented the inhibition of IFN-γ production and promoted the production of CXCL9 in CRC.

## Discussion

**Figure 6:**
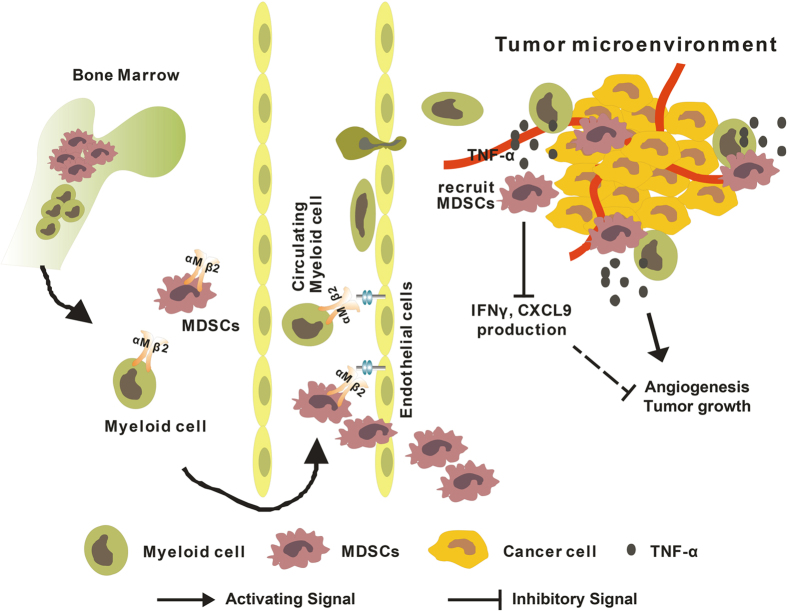
A schematic illustration of how CD11b deficiency might inhibit CRC tumor growth. The drawings in the figure were drawn by Q.Q.Z. using CorelDRAW.

In this study, we demonstrate an oncogenic role for CD11b during CRC tumorigenesis. Our findings demonstrated that CD11b expression on the myeloid cells promotes myeloid cell migration to the peripheral blood, spleen and tumor microenvironment. The myeloid cells also secrete cytokines, which promote MDSC recruitment to the tumor sites, further induce IFN-γ and CXCL9 production and activate Wnt/β-catenin signaling in CRC (Fig. 6).

Tumorigenesis is a complex process that involves many factors. A large number of reports indicated that chronic inflammation is an important factor for tumor development^43^. The tumor microenvironment is characterized by the chronic overexpression of inflammatory mediators that are produced by tumor-infiltrating immune cells, particularly bone marrow-derived cells. Therefore, the importance of the inflammatory tumor microenvironment cannot be overlooked. Recently, cytokines in the tumor microenvironment have been shown to contribute to angiogenesis, tumor cell proliferation, invasion and resistant to chemotherapy and radiotherapy. In CRC, tumor cells interact with cytokines in the tumor environment to activate the Wnt pathway^22^. Our work shows that the inhibition of myeloid cells infiltration into the CRC tumor environment can reduce the release of inflammatory factors and consequently activate the Wnt/β-catenin signaling pathway. The data generated in our current study further demonstrated the significance of the myeloid cells that infiltrate the tumor environment during tumorigenesis.

We found that the number of CD11b^+^ myeloid cells increased substantially in the bone marrow and spleens of the *Apc*^*Min/*+^ mice compared with the C57 mice. However, we did not observe a difference in the CD11b^+^ myeloid cells in the peripheral blood. The bone marrow and spleen are the important immune organs in which myeloid cells are generated and localized to activate an immune program. However, the peripheral blood is a part of the circulation system by which myeloid cells translocate from the central immune organs to the peripheral immune organ. Therefore, the differences in the CD11b^+^ myeloid cells may not be detected in the mobile phase of the peripheral blood. However, the peripheral blood of the *Apc*^*Min/*+^ mice tended to contain more CD11b^+^ myeloid cells than that of the C57 mice.

Tumor necrosis factor-α (TNF-α) is a key inflammatory cytokine that is primarily produced by myeloid cells^44^. However, it plays paradoxical roles in carcinogenesis. Reports indicated that TNF-α possesses both anti-tumor and pro-tumor activities^45^. It was reported that TNF-α can inhibit tumor growth in a breast cancer xenograft model^46^. Recently, a large number of studies demonstrated that TNF-α produced in tumor microenvironment may promote cancer development^47^,^48^,^49^,^50^. TNF-α produced by leukocyte was up-regulated in the colorectal carcinoma, and blocking the expression of TNF-α in mice can reduces colorectal carcinogenesis^51^. TNF-α is produced primarily by activated macrophages and also by other cell types including monocytes and lymphoid cells. We observed no differences between the macrophages from the *Apc*^*Min/*+^ mice and those in the *Apc*^*Min/*+^;*CD11b*^*−/−*^ mice (Supplementary Figure 5). However, the inhibition of TNF-α secretion in myeloid cells can significantly suppress HCT-116 cell proliferation. It is suggested that CD11b deficiency could inhibit the secretion of TNF-α by other type of myeloid cells, but not macrophage. Our results demonstrated that TNF-α, produced by myeloid cells, acts as a tumor-promoting factor that can significantly promote tumor growth in the *Apc*^*Min/*+^ mice.

CD11b, which is expressed on the surface of myeloid cells, has been widely implicated in mediating leukocyte adhesion and transendothelial migration^10^,^12^,^13^. CD11b inhibition can reduce myeloid cell recruitment in the tumor environment^11^. MDSCs are a population of bone marrow-derived immature myeloid cells, which constitute approximately 5% of the total cell population within CRC tumors^20^. However, granulocytic MDSC (Ly6G^+^Ly6C^low^) are often expanded in the tumor sites^16^,^19^. Our study has confirmed that CD11b deficiency cannot affect the production of CD45^+^ leukocytes and granulocytic Ly6G^+^Ly6C^low^ MDSCs in the bone marrow. However, the number of the Ly6G^+^Ly6C^low^ subset of MDSCs was dramatically decreased in the peripheral blood, spleen and tumor sites. Our work suggests that CD11b may not mediate the production of these cells, but only regulates the transendothelial migration of the Ly6G^+^Ly6C^low^ subset MDSCs. These data are consistent with previous reports showing that CD11b deficiency is characterized by defects in leukocyte adhesion and migration across the endothelium^52^.

In the tumor, CD11b inhibition led to a significant enhancement of the tumor response to irradiation by suppressing vasculogenesis, with no effect on non-irradiated tumors from hypopharyngeal carcinoma cells that were transplanted into immunodeficient mice^11^. However, our data showed that CD11b deficiency can decrease the number of myeloid cells in tumor environment, and thereby inhibit angiogenesis and tumor growth over the protracted course (15 weeks) of tumor development in *Apc*^*Min/*+^ mice under normal conditions, which were not consistent with previously published results^11^. MDSCs suppress T cell function, which contributes to tumor growth. The defective T cells evoke the functional disruption of myeloid cells in the immunodeficient mice with transplanted tumor. Our work was performed in mice with a normal immune system, which may not affect MDSC suppression of T cell function. In support of our data, previous report indicated that myeloid cells promote tumor growth by stimulating tumor angiogenesis and suppressing tumor immunity^17^. The data generated in our current study not only support the published data that CD11b deficiency can reduce myeloid cell recruitment in tumor environment, but also indicate that CD11b deficiency is likely to inhibit angiogenesis and tumor growth in CRC tumor-bearing mice.

In summary, our data have demonstrated that CD11b is critically involved in the transendothelial migration of bone marrow-derived immune cells to the tumor sites, resulting in intestinal tumorigenesis. Moreover, CD11b may serve as a potential biomarker for therapy of CRC treatment.

## Additional Information

**How to cite this article**: Zhang, Q.-Q. *et al.* CD11b deficiency suppresses intestinal tumor growth by reducing myeloid cell recruitment. *Sci. Rep.* **5**, 15948; doi: 10.1038/srep15948 (2015).

## Supplementary Material

Supplementary Information
