# Supplementary material for: CD11b deficiency suppresses intestinal tumor growth by reducing myeloid cell recruitment
Source: Sci Rep. 2015 Nov 3;5:15948. doi: 10.1038/srep15948 (PMC4630647; doi:10.1038/srep15948)
Supplement: Supplementary Information [file srep15948-s1.pdf]

Title: CD11b deficiency suppresses intestinal tumor growth by reducing myeloid cell recruitment

Authors and affiliation:

Qian-Qian Zhang[1]†, Xi-Wen Hu[1]†, Yi-Long Liu[1], Zhi-Jin Ye[1], Yi-He Gui[1], Da-Lei Zhou[1], Cui-Ling Qi[1], Xiao-Dong He[1], Honglin Wang[2]*, Li-Jing Wang[1]*

1. Vascular Biology Research Institute, Guangdong Pharmaceutical University, Guangzhou 510006, China
2. Institute of Immunology, Institute of Medical Sciences, Shanghai Jiao Tong University School of Medicine, Shanghai 200025, China

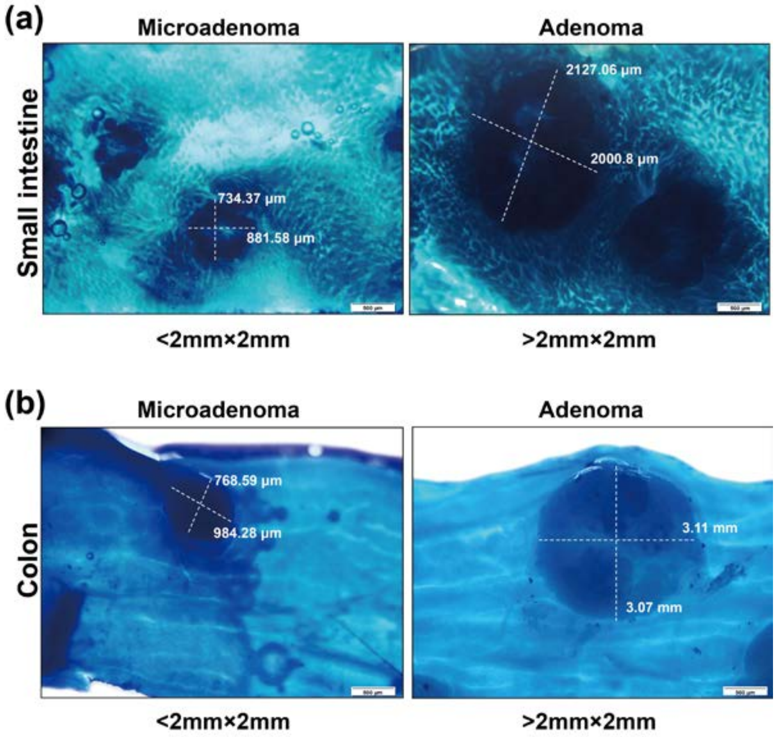

Supplementary Figure 1. Classification of intestinal tumor. The intestinal (**A**) and colonic (**B**) tumors were classified into 2 types: microadenomas and adenomas. Microadenomas were defined as on greater than 2 mm × 2 mm in size, and the adenomas were greater than 2 mm × 2 mm in size detected under microscope.

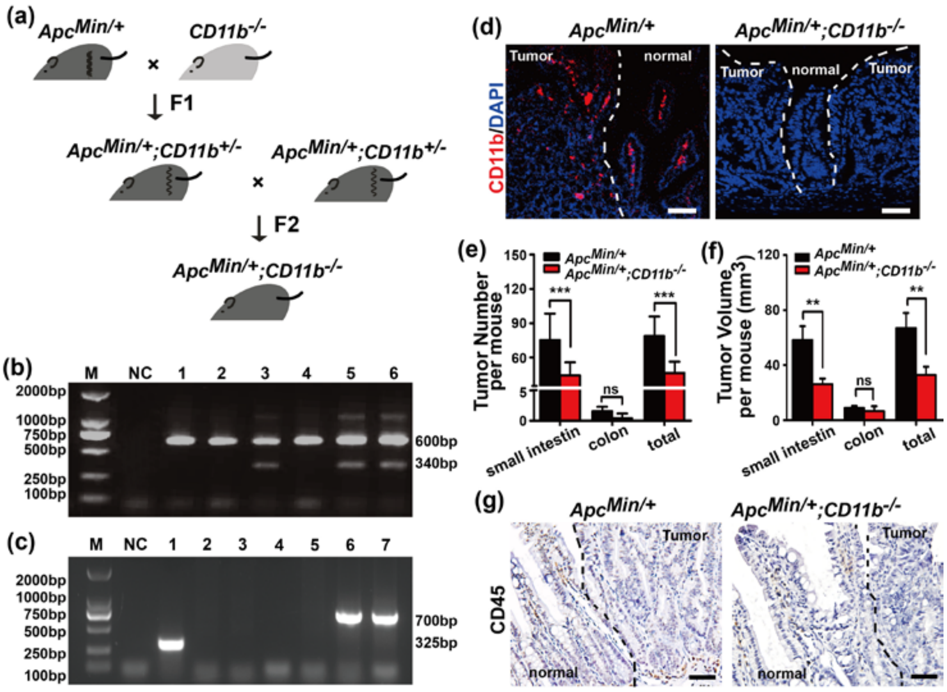

Supplementary Figure 2. Construction of Slit2-overexpressing mice. A schematic diagram of generating *Apc*$^{Min/+}$;*CD11b*$^{-/-}$ experimental mice carrying both the *CD11b* gene deficiency and Apc mutant allele is shown in panel **A**. PCR screening of *Apc*$^{Min/+}$;*CD11b*$^{-/-}$ mice using primers that detect both wild-type and mutant alleles of *Apc* (**B,** *Apc* wild-type and mutant alleles, 619 bp and 331 bp; *Apc* wild-type allele, 619 bp), and both wild-type and deficiency alleles of *CD11b* (**C**, *CD11b* wild-type allele, 325 bp; *CD11b* deficiency allele, 700 bp). M: molecular weight marker D2000; NC: negative control. The CD11b+ myeloid cells infiltrating was detected by IF in the tumor tissues and adjacent normal tissues of the *Apc*$^{Min/+}$ mice and *Apc*$^{Min/+}$;*CD11b*$^{-/-}$ mice (**D**). CD11b deficiency significantly inhibited the number (**E**) and size (**F**) of the tumors in small intestine but not colon in the *Apc*$^{Min/+}$ mice. The CD45+ leukocytes infiltration were suppressed in the tumor sites, but were not inhibited in the adjacent normal tissues from the *Apc*$^{Min/+}$;*CD11b*$^{-/-}$ mice compared with the *Apc*$^{Min/+}$ mice (**G**). Scale bars: 100 μm. All the drawings in figure (a) were drawn by Q.Q.Z. using

CorelDRAW.

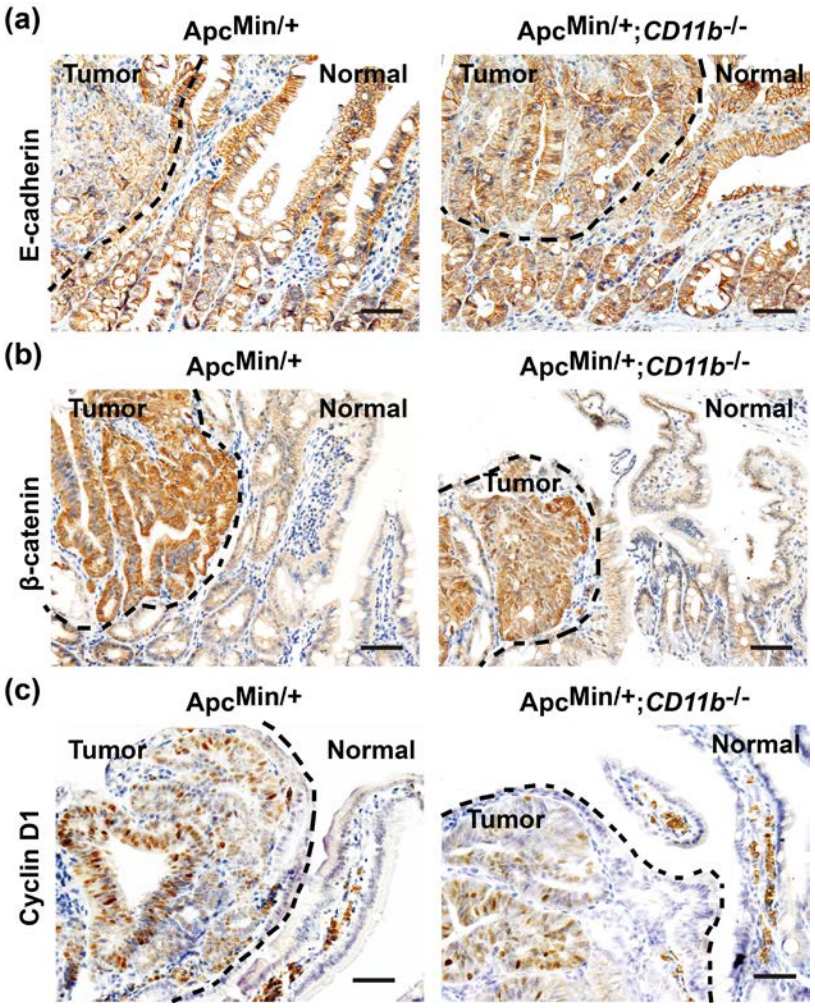

Supplementary Figure 3. CD11b deficiency inactivated the Wnt/β-catenin pathway in tumor tissues. The expression of E-cadherin (**A**), β-catenin (**B**) and cyclin D1 (**C**) was examined in the tumor tissues and adjacent normal tissues of the $Apc^{Min/+}$ mice and $Apc^{Min/+}$*;CD11b*$^{-/-}$ mice by IHC. Scale bars: 100 μm.

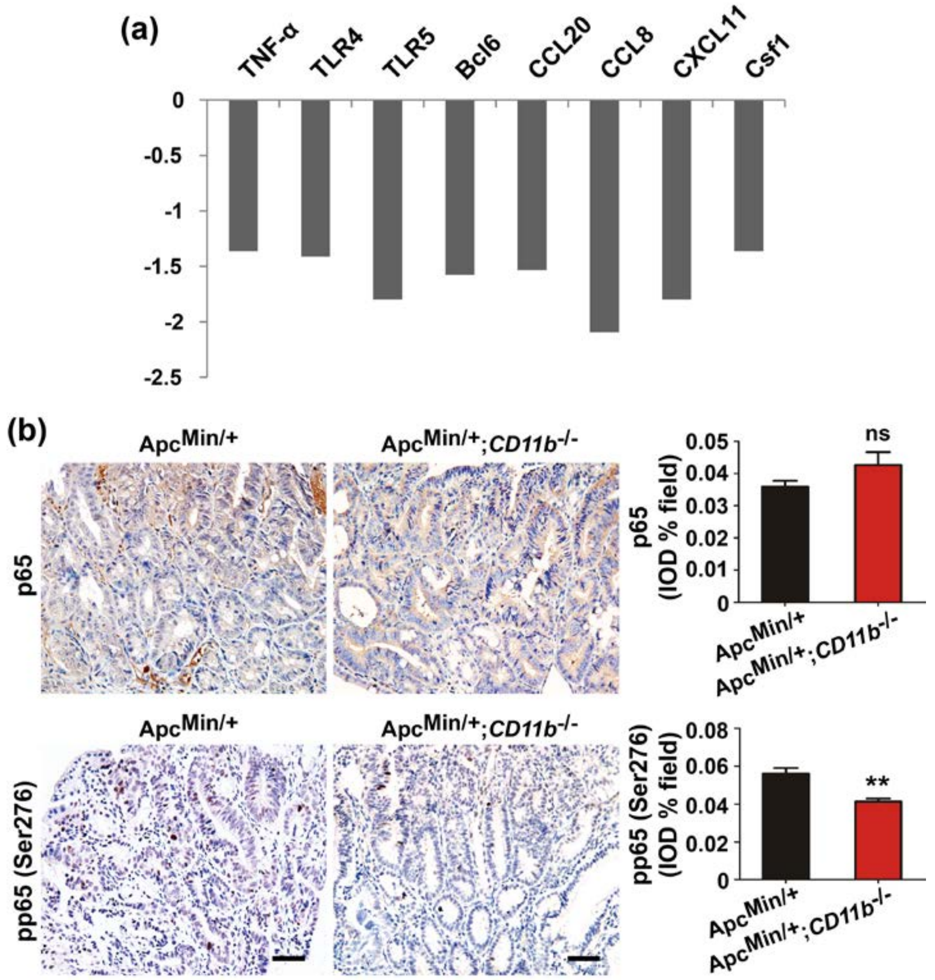

Supplementary Figure 4. The differential expression cytokines and NF-κB signaling may affected by CD11b+ myeloid cells. Total RNA was extracted from spleen, and cytokines that were potentially regulated by CD11b+ myeloid cells were identified by qRT-PCR array. The histogram shows the cytokines with more than two-fold change in spleen (**A**). The expression of p65 and pp65 (Ser276) in tumor tissues of the $Apc^{Min/+}$ mice and $Apc^{Min/+}$;$CD11b^{-/-}$ mice was detected by IHC (**B**). The results of are representative of 11 independent mice (all mice were 16 –weeks -old). The statistical data are expressed as the mean ± S.D.. ns: $P > 0.05$, **: $P < 0.01$. Scale bars: 50 μm.

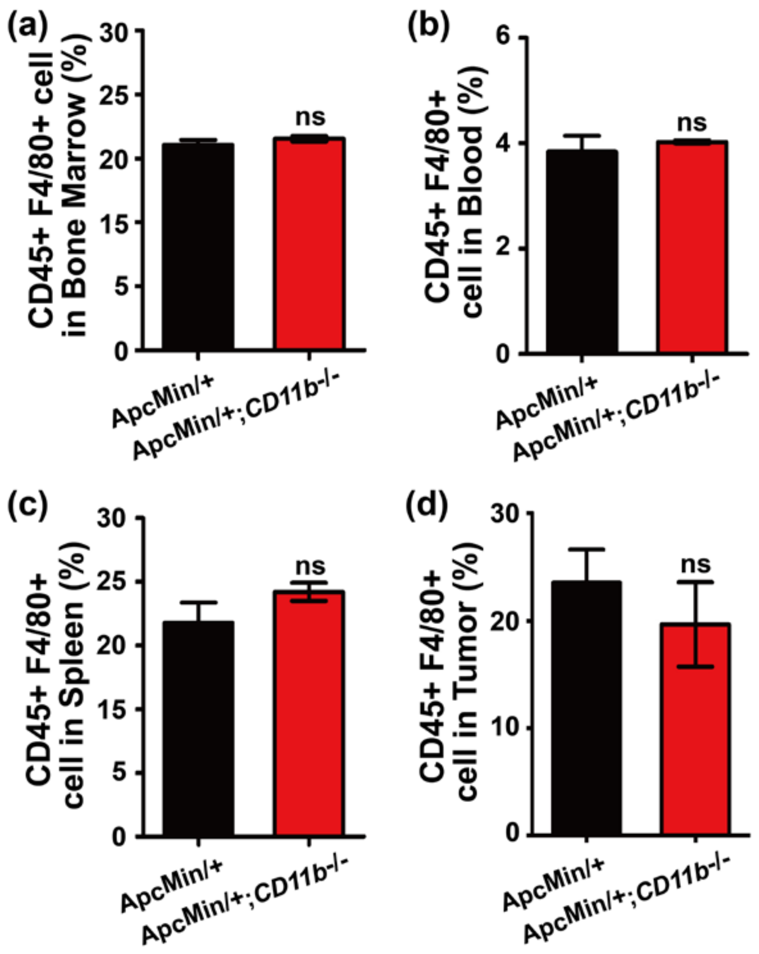

Supplementary Figure 5. CD11b deficiency did not affect macrophage recruitment in the tumor environment. The number of the macrophage (gated on $CD45^+$ cells) in the bone marrow (**A**), blood (**B**), spleen (**C**) and tumor tissues (**D**) of the *Apc*$^{Min/+}$ mice and *Apc*$^{Min/+}$*;CD11b*$^{-/-}$ mice was measured by FACS. The results represent of 11 independent mice (all mice were 16 –weeks -old). The statistical data are expressed as the means ± S.D.. ns: $P > 0.05$.
